# An Azo‐Based Electrode for All‐Around High‐Performance Flexible Supercapacitors

**DOI:** 10.1002/smsc.202200101

**Published:** 2023-03-09

**Authors:** Haoxiang Zhang, Minyong Du, Xinxin Xing, Hui Wang, Kai Wang, Shengzhong (Frank) Liu

**Affiliations:** ^1^ Dalian National Laboratory for Clean Energy Dalian Institute of Chemical Physics Chinese Academy of Sciences Dalian Liaoning 116023 China; ^2^ Center of Materials Science and Optoelectronics Engineering University of Chinese Academy of Sciences Beijing 100049 China; ^3^ Key Laboratory of Applied Surface and Colloid Chemistry Ministry of Education Shaanxi Key Laboratory for Advanced Energy Devices Shaanxi Engineering Lab for Advanced Energy Technology Institute for Advanced Energy Materials School of Materials Science and Engineering Shaanxi Normal University Xi'an 710119 China

**Keywords:** energy density, flexible electronics, organic electrodes, photo-rechargeable supercapacitors

## Abstract

The photo‐rechargeable supercapacitor enables the self‐powering of flexible wearable electronics. However, flexible wearable electronics require supercapacitors not only with excellent flexibility but also with high energy density. P‐diaminoazobenzene (P‐Azo) as a new type of organic electrode material with N=N is directly connected to the benzene ring and forms a large π‐conjugated system, which makes it have a lower lowest unoccupied molecular orbital (LUMO) energy level, is beneficial to transfer of electrons, and increases the conductivity of organic molecules. In addition, N=N can realize the transfer of two electrons, which makes P‐Azo have a higher energy density. Asymmetric flexible supercapacitors are fabricated by assembling P‐Azo, activated carbon, and an adhesive electrolyte, with 425.2 mW h cm^−2^ (55.19 Wh kg^−1^) energy density at a power density of 80 mW cm^−2^ (10.38 W kg^−1^), and 90.7% capacitance retention after 80 000 cycles of bending. In this work, supercapacitors and perovskite submodules are coupled to prepare a photo‐rechargeable supercapacitor to achieve a 7% overall energy‐conversion efficiency. Therefore, this supercapacitor paves a practical route for powering future wearable electronics.

## Graphical abstract

High‐energy‐density, high‐power‐density, and fatigue‐resistant flexible azo‐based supercapacitor and perovskite submodules are coupled to prepare a photo‐rechargeable supercapacitor to achieve a 7% overall energy conversion efficiency.

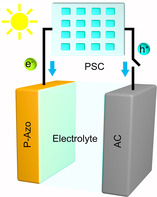

## 1 Introduction

Light‐charging self‐powered systems can timely convert solar energy into electric energy, which can be used by wearable devices and meet the demand of electricity consumption.^[^ ^1^ ^]^ By coupling a solar cell and an electrochemical energy‐storage device supercapacitor, the prepared device is a photo‐rechargeable supercapacitor,^[^ ^2^ ^]^ which can effectively alleviate the instability of solar cells during use and has great application potential in smart electronic products, etc.^[^ ^3^ ^]^ In terms of energy harvesting, perovskite solar cells (PSCs) in particular have the characteristics of high extinction coefficient, low exciton‐binding energy, and long carrier‐diffusion distance, so they have become an important research topic.^[^ ^4^ ^]^ The energy‐storage devices are supercapacitors,^[^ ^5^ ^]^ and they have high power density, excellent rate performance, and good cycle stability,^[^ ^6^ ^]^ which is conducive to the transfer of charge from the solar cells to the supercapacitors and improves photoelectric performance.^[^ ^7^ ^]^ Although supercapacitors have high power density, their energy density still needs to be further improved to meet the requirements of practical applications.^[^ ^8^ ^]^

The energy density of a supercapacitor is *E = *(*CV* ^2^)/2, where *C* is the capacitance of the supercapacitor, and *V* is the electrochemical window, so improving the electrochemical window and specific capacity of the supercapacitor can effectively improve the energy density of the supercapacitor. The electrochemical window can be improved by fabricating asymmetric supercapacitors.^[^ ^9^ ^]^ For improving the specific capacity of supercapacitors, the conventional strategy is to combine high‐conductivity carbon nanotubes, grapheme,^[^ ^10^ ^]^ or MXene^[^ ^11^ ^]^ with pseudocapacitive materials, where the specific capacity of the electrode can be improved by utilizing the complementary strategies of the two materials.^[^ ^12^ ^]^ In addition, flexible wearable electronics require supercapacitors not only with high energy density but also with flexibility.^[^ ^13^ ^]^

Organic electrode materials have the advantages of abundant sources,^[^ ^14^ ^]^ low costs, high theoretical specific capacity,^[^ ^15^ ^]^ processabilities, and fast ion‐diffusion rate,^[^ ^16^ ^]^ and are potential materials for the fabrication of high‐energy‐density flexible supercapacitors (FSCs). According to the different redox‐active sites, there are mainly four kinds of organic electrode materials at present, namely, organic sulfides (S–S), organic radicals (–O), imine compounds (C=N), and carbonyl compounds (C=O).^[^ ^17^ ^]^ S–S has a higher theoretical specific capacity, but the small organic sulfide molecules are easily soluble in the electrolyte during the redox process, reducing the electrical properties of the electrodes.^[^ ^18^ ^]^ The most commonly used organic radical is 2,2',6,6'‐tetramethylpiperidine‐1‐oxyl radical (TEMPO), which has a higher rate due to its high conductivity. However, there are fewer active sites in the organic radical molecule, so its energy density is lower.^[^ ^19^ ^]^ The current organic electrode materials mainly focus on imines and carbonyl compounds, through the reversible electrochemical reaction of C=N or C=O with cations, to achieve charge storage and release. The flexible free‐standing supercapacitor was prepared by 2,4,6‐trimethoxy‐1,3,5‐benzene‐tricarbaldehyde and 2,6‐diaminoanthraquinone Schiff's base reaction, the device had an areal specific capacity of 1600 mF cm^−2^ at a current density of 3.3 mA cm^−2^.^[^ ^20^ ^]^ The flexible porous anthraquinone polymer prepared by nucleophilic reaction of 2,6‐diaminoanthraquinone and aryl bromides has a mass specific capacity of 576 F g^−1^ at a current density of 1 A g^−1^.^[^ ^21^ ^]^

In addition to the aforementioned four types of organic electrode materials, azo compounds, as a new type of organic electrode materials, whose active sites N=N can interact with metal ions through reversible interactions that can achieve high‐capacity charge storage.^[^ 15, 17 ^]^ Among many azo compounds, P‐diaminoazobenzene (P‐Azo) with an active site (N=N) is directly connected to the benzene ring and forms a large π‐conjugated system, which makes it have a lowest unoccupied molecular orbital (LUMO), which is beneficial to electrons transfer and increases the conductivity of organic molecules.^[^ ^22^ ^]^ In addition, the active site (N=N) of the P‐Azo can realize the transfer of two electrons, which makes the P‐diaminoazobenzene to have a higher energy density. Here, for the first time, we used P‐Azo as electrode materials and successfully fabricated flexible supercapacitors with high energy density, high power density, and fatigue resistance. Based on the aforementioned performance of these supercapacitors, they are coupled with PSC to prepare a photo‐rechargeable supercapacitor. Utilizing photo‐generated charge storage in PSC via N=N redox, the prepared photo‐rechargeable supercapacitor achieves a 7% overall energy‐conversion efficiency.

## 2 Results and Discussion

### 2.1 Electrochemical Performance of P‐Azo

**Figure 1:**
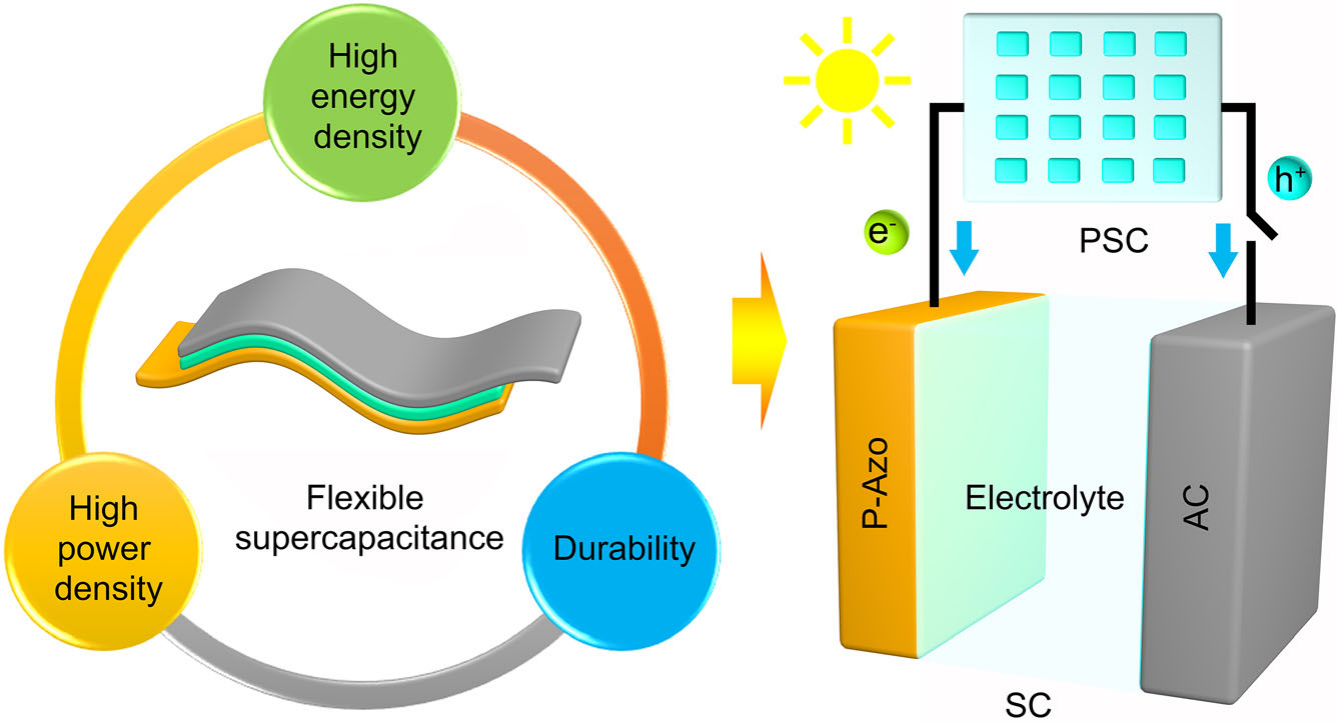
Schematic illustration of the photo‐rechargeable supercapacitors.

The N=N in p‐diaminoazobenzene (P‐Azo) acts as an active site for redox reactions.^[^ ^18^ ^]^ Differing from anthraquinone organic molecules with one electron transfer per active site, the azo molecules perform two electron transfers per redox‐active site. The theoretical specific capacity of the P‐Azo is calculated using Equation (1)

(1) $$Q=\frac{nF}{3.6\times M_{w}}$$

where *n* is the number of electrons transferred by the redox reaction, *F* is the faraday constant, and *M*_w_ is the molecular weight of the organic molecule. According to Equation (1), the theoretical specific capacity of P‐Azo is 252.55 mA h g^−1^. This high specific capacity of P‐Azo was used along with activated carbon to assemble high‐energy‐density, high‐power‐density, and fatigue‐resistant all‐around high‐performance asymmetric flexible supercapacitors, which were fabricated into photo‐rechargeable supercapacitors (**Figure** **1**).

Flexible electrodes were prepared by coating P‐Azo with a carbon cloth surface. Scanning electron microscope (SEM) images (Figure S1a, Supporting Information) and transmission electron microscope (TEM) images (Figure S1b, Supporting Information) showed that P‐Azo was coated on the surface of carbon cloth. The energy‐dispersive X‐ray spectroscopy (EDX) mapping images showed that C and N were uniformly distributed on the electrode surface (Figure S1c, Supporting Information), and X‐ray photoelectron spectroscopy (XPS) spectra also confirmed the existence of C and N (Figure S2, Supporting Information).

**Figure 2:**
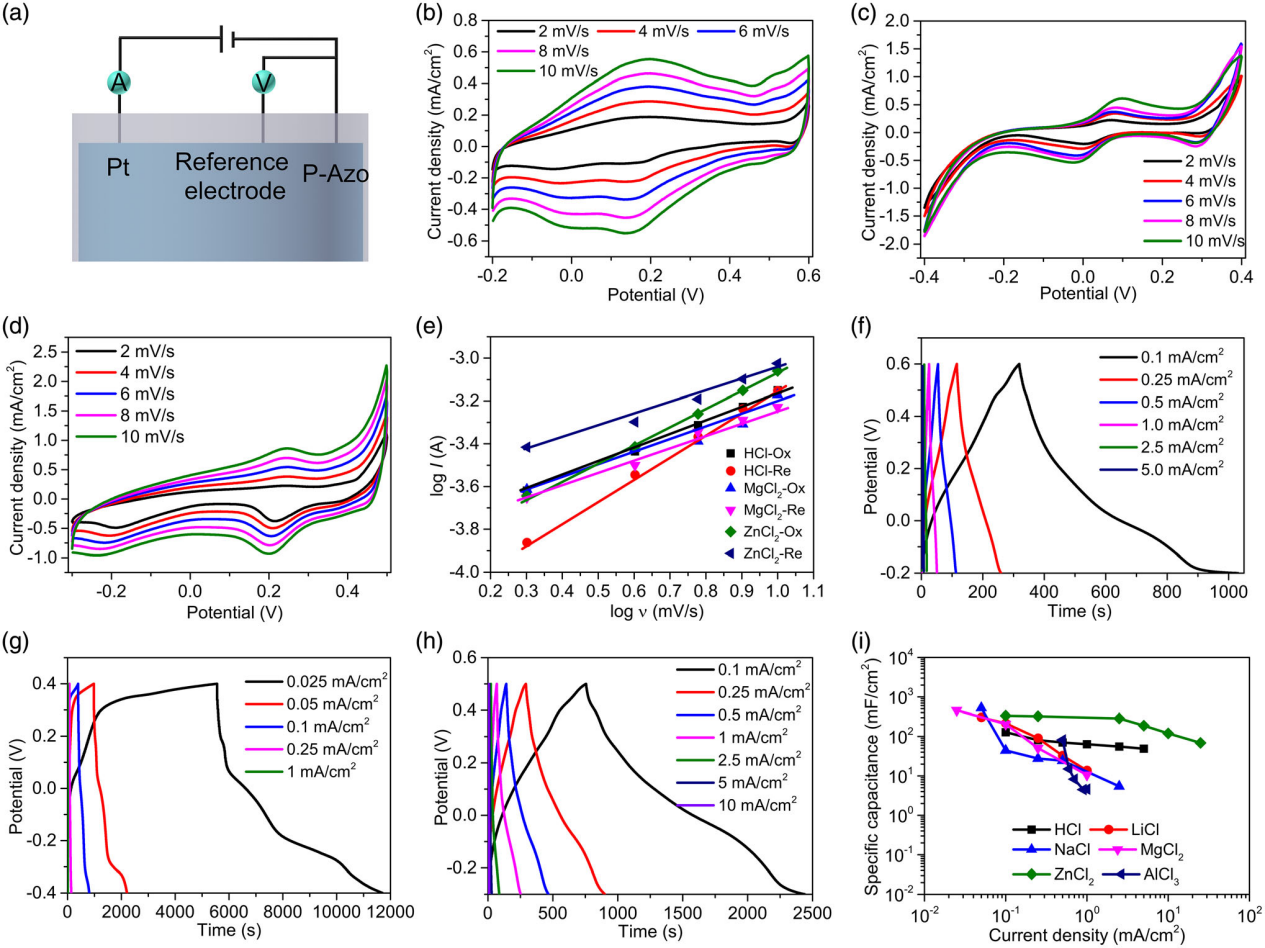
Electrochemical performance of electrodes in different electrolytes. a) Schematic diagram of the three‐electrode test structure. b–d) CV curves of P‐Azo in 1 mol L^−1^ HCl (b), MgCl_2_ (c), and ZnCl_2_ (d) solution. e) Fitting curves of the *P*‐Azo oxidation peak current versus scan rate. f–h) Galvanostatic charge–discharge (GCD) curves of P‐Azo in 1 mol L^−1^ HCl (f), MgCl_2_ (g), and ZnCl_2_ (h) solution. i) The areal specific capacity of P‐Azo in different electrolytes as a function of current density.

In the electrolyte, different cations have different physical and chemical properties, such as binding energy, ionic radius, number of transferred electrons, etc., which affect the electrical properties such as redox potential and specific capacity. The cyclic voltammetry (CV) curves of P‐Azo in 1 mol L^−1^ HCl, LiCl, NaCl, MgCl_2_, ZnCl_2_, and AlCl_3_ electrolytes were generated (**Figure** **2**a). In the different electrolytes, P‐Azo shows different oxidation peaks, which are located at 0.197 (Figure 2b), −0.012 (Figure S3a, Supporting Information), 0.017 (Figure S3b, Supporting Information), 0.098 (Figure 2c), 0.247 (Figure 2d), and −0.35 V (Figure S3c, Supporting Information). It is worth mentioning that P‐Azo also showed obvious reduction peaks in HCl, MgCl_2_, and ZnCl_2_, indicating that N=N undergoes reversible redox reactions in these solutions. However, in Li^+^, Na^+^, and Al^3+^ solutions, there is no obvious reduction peak, which indicates the reversibility of N=N is poor in the aqueous solutions of these three ions.

The oxidation peak current of P‐Azo showed an increasing trend with the increase in scan rate. The surface capacitive‐ and diffusion‐controlled contributions of P‐Azo during charge and discharge can be calculated from curve fits using a power law^[^ ^23^ ^]^

(2) log i=b log v+log a

where *i* is the peak current and *v* is the scan rate. The value of *b* is obtained by plotting log*i* versus log*v* and calculating the slope. If *b* is close to 0.5, it means that the charge–discharge process is mainly controlled by diffusion; if *b* is close to 1, the charge–discharge process is mainly controlled by surface diffusion. Figure 2e shows the regression equation fitted to log *i* versus log *v* for HCl, MgCl_2,_ and ZnCl_2_ solutions, and the slopes, *b*, have been calculated (Table S1, Supporting Information). It can be observed that P‐Azo electrodes have different slopes in different electrolytes. The *b* values of the P‐Azo electrode at the oxidation and reduction peaks in MgCl_2_ are 0.57 and 0.62, respectively, indicating that the redox reaction of P‐Azo electrodes is mainly controlled by diffusion in the MgCl_2_ solution. In the HCl and ZnCl_2_ solutions, the P‐Azo electrodes exhibit larger *b* values, indicating that the diffusion‐controlled contribution of the electrode in these two solutions is gradually weakened. The previous results show that the nitrogen anions formed after oxidation of N=N have different binding forces with different cations.^[^ ^24^ ^]^ The electrochemical impedance spectroscopy (EIS) curves show that P‐Azo has different diffusion resistances in different solutions (Figure S4, Supporting Information), which further confirms that the P‐Azo has different binding forces in different cation solutions.

To quantitatively calculate the proportion of capacitive control and diffusion control, the capacitive contribution rate of the electrode is calculated using Equation (3)^[^ ^23^ ^]^

(3) $$i=k_{1}v+k_{2}v^{1/2}$$

where *k*_1_ *v* is the capacitive contribution current and *k*_2_ *v* ^1/2^ is the diffusion contribution current. The capacitance contribution ratios of the P‐Azo were calculated at the scan rate of 2–10 mV s^−1^. At the scan rate of 2 mV s^−1^, the capacitance contribution of the P‐Azo electrodes in the MgCl_2_ solution was only 29% (Figure S5a, Supporting Information), which was lower than those in the HCl solution (48.4%) (Figure S5b, Supporting Information) and ZnCl_2_ solution (37.5%) (Figure S5c, Supporting Information), further confirming that the redox reaction of the electrode in MgCl_2_ solution is mainly controlled by diffusion. At an increased electrode scan rate of 1000 mV s^−1^, the shape of the CV curve is still well maintained (Figure S6, Supporting Information), indicating that P‐Azo electrodes have fast ion‐diffusion and charge‐storage capability.^[^ ^25^ ^]^

Galvanostatic charge–discharge (GCD) curves were obtained to evaluate the energy‐storage capability of the P‐Azo in different solutions. At various charge and discharge current densities of the electrode, the GCD curves of the P‐Azo were generated in 1 mol L^−1^ HCl (Figure 2f), LiCl (Figure S7a, Supporting Information), NaCl (Figure S7b, Supporting Information), MgCl_2_ (Figure 2g), ZnCl_2_ (Figure 2h), and AlCl_3_ (Figure S7c, Supporting Information) solutions. The P‐Azo has a longer charge–discharge time in MgCl_2_ and ZnCl_2_, indicating that N=N has a better storage capacity for Mg^2+^ and Zn^2+^. In MgCl_2_ solution, the electrode has an areal specific capacity of 456.98 mF cm^−2^ at a current density of 0.25 mA cm^−2^ (Figure 2i), which is higher than the 333.87 mF cm^−2^ in ZnCl_2_ solution, 128.82 mF cm^−2^ in HCl solution, 90.64 mF cm^−2^ in LiCl solution, and 27.53 mF cm^−2^ in NaCl solution. This result shows that, compared with other kinds of electrolytes, P‐Azo has a higher charge‐storage capacity in MgCl_2_.

### 2.2 Electrochemical Reaction Mechanism of Organic Electrodes

**Figure 3:**
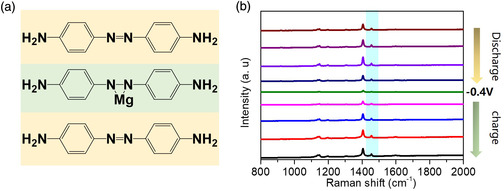
a) Redox mechanism of P‐Azo in MgCl_2_ solution, b) redox mechanism of P‐Azo in MgCl_2_ solution.

In the Mg^2+^ electrolyte, the adsorption and desorption of ions occur through the redox of the N=N bond. To analyze the structural changes of P‐Azo during the charging and discharging process, ex situ Raman spectroscopy was performed to investigate the N=N redox reaction. When the P‐Azo gains two electrons during discharge and is opened to form a nitrogen anion and a coordination bond with Mg^2+^ (**Figure** **3**a), the N=N Raman absorption peak at 1455 cm^−1^ gradually disappears (Figure 3b).^[^ ^14^ ^]^ When the electrode loses electrons after charge, the absorption peak reappears at 1455 cm^−1^ and the peak intensity gradually increases, indicating that the N=N structure is formed again, which is consistent with previous test results.^[^ ^26^ ^]^

To further prove that the electrons of N=N become nitrogen negative ions during the charging and discharging process, the ultraviolet spectrum of the MgCl_2_ electrolyte was acquired after 500 cycles of charge and discharge, and for comparison, the ultraviolet spectrum of P‐Azo in aqueous solution (10^−5^ mol L^−1^) was also obtained. Among them, P‐Azo has a strong π–π* transition absorption peak at 392 nm in a 10^−5^ mol L^−1^ aqueous solution (Figure S8, Supporting Information),^[^ ^27^ ^]^ while the π–π* transition absorption peak intensity in the electrolyte after 500 charge and discharge cycles is significantly reduced, indicating that the organic electrode molecules dissolved in MgCl_2_ mainly exist in the form of nitrogen anions.

### 2.3 Fabrication and Performance Testing of the FSCs

**Figure 4:**
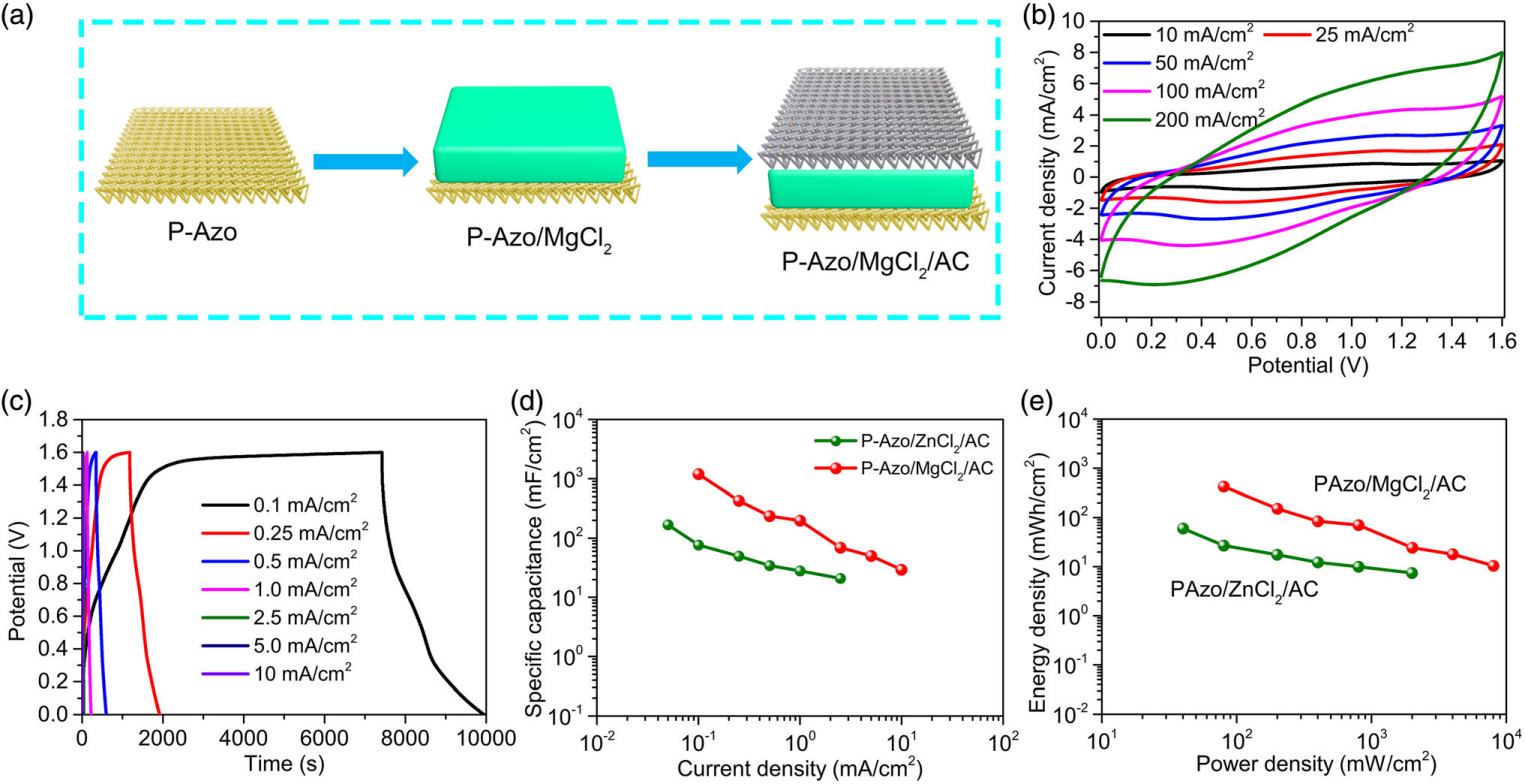
Electrochemical performance of supercapacitors. a) Schematic diagram of the fabrication of the supercapacitors. b) CV curves and c) GCD curves of a P‐Azo/MgCl_2_/AC FSC. d) The areal specific capacity of a P‐Azo/MgCl_2_/AC FSC and a P‐Azo/ZnCl_2_/AC FSC as a function of current density, and e) the energy density as a function of power density for various reported supercapacitors.

To meet the application needs of flexible supercapacitors in flexible wearable electronic products, P‐Azo was used as the negative electrode, activated carbon (AC) was used as the positive electrode, and Mg^2+^ or Zn^2+^ polyacrylamide ionic hydrogel was used as the gel electrolyte to prepare flexible all‐solid‐state supercapacitors (P‐Azo/MgCl_2_/AC FSC and P‐Azo/ZnCl_2_/AC FSC) (**Figure** **4**a). By adding Mg^2+^ or Zn^2+^ to the hydrogel in polyacrylamide, the prepared gel electrolyte has adhesion and can firmly adhere to the positive and negative electrodes,^[^ ^28^ ^]^ reducing the contact resistance between the gel electrolyte and the positive and negative electrodes. The EIS curves show that the internal resistances (*R*_s_) of the P‐Azo/MgCl_2_/AC FSC and the P‐Azo/ZnCl_2_/AC FSC are 8.2 and 8.7 Ω (Figure S9, Supporting Information), respectively, confirming that the gel electrolyte has good contact with the positive and negative electrodes of the supercapacitors. The energy‐storage performance of these flexible supercapacitors was evaluated using CV curves and GCD curves. The P‐Azo/MgCl_2_/AC FSC (Figure 4b) and P‐Azo/ZnCl_2_/AC FSC (Figure S10a, Supporting Information) still showed good morphology retention at scan rates of 10–200 mV s^−1^, indicating good diffusivity of Mg^2+^ and Zn^2+^ in the positive and negative electrodes^[^ ^29^ ^]^ and that the strong adhesion at the interfaces formed by the gel electrolyte and the positive and negative electrodes of the supercapacitor promotes the transport of ions at the electrode and gel interfaces.

The P‐Azo/MgCl_2_/AC FSC can be charged and discharged in the current density range of 0.1–10 mA cm^−2^ (Figure 4c), which is wider than that of the P‐Azo/ZnCl_2_/AC FSC (Figure S10b, Supporting Information). The areal specific capacities of the supercapacitors can be calculated from the GCD curves. At a current density of 0.1 mA cm^−2^, the P‐Azo/MgCl_2_/AC FSC has an areal specific capacity of 1195.88 mF cm^−2^, higher than that of the P‐Azo/ZnCl_2_/AC FSC (76.27 mF cm^−2^). The areal specific capacity of the P‐Azo/MgCl_2_/AC FSC is consistently higher than that of the P‐Azo/ZnCl_2_/AC FSC with increasing current density (Figure 4d). In addition, the areal specific capacity of the P‐Azo/MgCl_2_/AC FSC is also higher than those of similar supercapacitors reported so far, fibrous P3MT/HACNT//HACNT (640 mF cm^−2^),^[^ ^29^ ^]^ PEDOT:PSS//PEDOT:PSS (8.45 mF cm^−2^),^[^ ^30^ ^]^ CNC/PEDOT:PSS (380.5 mF cm^−2^),^[^ ^31^ ^]^ GMPH7//GMPH7 (584.7 mF cm^−2^),^[^ ^32^ ^]^ P(TEBTT/EDOT)//P(TEBTT/EDOT) (443.8 mF cm^−2^),^[^ ^33^ ^]^ Ti_3_C_2_T_*x*_‐HA//C‐MnO_2_ (790.9 mF cm^−2^), Ti_3_C_2_T_*x*_‐HA//C‐MnO_2_ (660.9 mF cm^−2^),^[^ ^34^ ^]^ and PPy/l‐Ti_3_C_2_//PPy/l‐Ti_3_C_2_^[^ ^35^ ^]^ polymetallic oxide–graphene (862.72 mF cm^−2^).^[^ ^36^ ^]^

The P‐Azo/MgCl_2_/AC FSC has a higher energy density of 425.2 mW h cm^−2^ (55.19 Wh kg^−1^) at a power density of 80 mW cm^−2^ (10.38 W kg^−1^), and 10.44 mW h cm^−2^ (1.35 Wh kg^−1^) energy density at a power density of 8000 mW cm^−2^ (1038.34 W kg^−1^), higher than P‐Azo/ZnCl_2_/AC FSC electrochemical performance, with 59.87 mW h cm^−2^ (7.99 Wh kg^−1^) energy density at 40 W cm^−2^ (5.34 W kg^−1^) power density, 7.43 mW h cm^−2^ (0.99 Wh kg^−1^) energy density at a power density of 1995.42 W cm^−2^ (266.53 W kg^−1^). These values are higher than the power density and energy density of similar supercapacitors reported so far (Figure 4e), such as PEDOT:PSS/ferritin//MWNT SC (0.00082 mW h cm^−2^, 0.15 mW cm^−2^),^[^ ^37^ ^]^ AQS@rGO//rGO SC (3420.0 mW h cm^−2^, 29.2 mW cm^−2^),^[^ ^38^ ^]^ tetra‐aniline SC (0.8 mW h cm^−2^, 8.2 mW cm^−2^),^[^ ^39^ ^]^ GH‐DN//rGO‐NDI SC (0.6 mW h cm^−2^, 26.3 mW cm^−2^),^[^ ^40^ ^]^ P3MT/HACNT//HACNT (1.75 mW h cm^−2^, 1.08 mW cm^−2^),^[^ ^29^ ^]^ PEDOT:PSS//PEDOT:PSS SC (0.4 mW h cm^−2^, 1.63 mW cm^−2^),^[^ ^30^ ^]^ GMPH7//GMPH7 SC (0.5 mW h cm^−2^, 0.08 mW cm^−2^),^[^ ^32^ ^]^ 3D MXene inks SC (0.00011 mW h cm^−2^, 0.158 mW cm^−2^),^[^ ^41^ ^]^ Lig/PANI//FGH/FCC SC (1 mW h cm^−2^, 0.16 mW cm^−2^),^[^ ^42^ ^]^ Co_9_S_8_@PPy@NiCo‐LDH //AC (0.8 mW h cm^−2^, 0.13 mW cm^−2^),^[^ ^43^ ^]^ PANI/rGO//PTCA/rGO (14 mW h cm^−2^, 520 mW cm^−2^),^[^ ^44^ ^]^ and PEDOT:PSS cellulose//polyester cloth SC (1.36 Wh kg^−1^, 329.7 W kg^−1^).^[^ ^45^ ^]^

**Figure 5:**
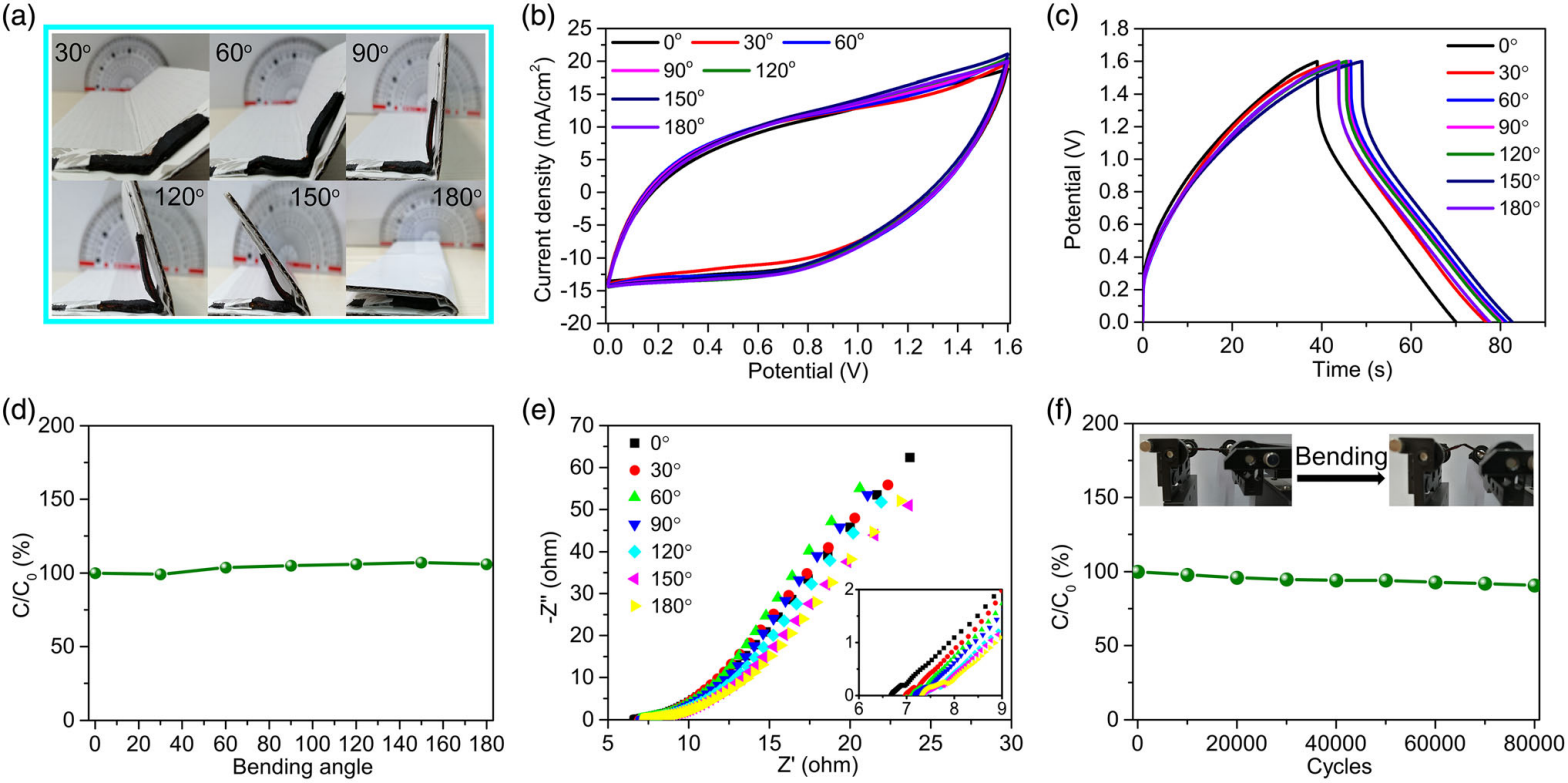
Electrochemical stability of supercapacitors. a) Photos of the P‐Azo/MgCl_2_/AC FSC bent at various angles. b) CV curves and c) GCD curves of P‐Azo/MgCl_2_/AC FSC at different bending angles. d) Capacitance retention of a P‐Azo/MgCl_2_/AC FSC as a function of bending angle. e) Electrochemical impedance spectroscopy (EIS) curves at different bending angles. f) Capacitance retention of a P‐Azo/MgCl_2_/AC FSC after 80 000 bending cycles.

To study the flexibility of the P‐Azo/MgCl_2_/AC FSC, the electrochemical performance of the supercapacitor was tested at bending angles of 0°, 30°, 60°, 90°, 120°, 150°, and 180° (**Figure** **5**a). The CV curves (Figure 5b) and GCD curves (Figure 5c) of a P‐Azo/MgCl_2_/AC FSC at different bending angles did not change significantly, and the calculated capacitance retention of the P‐Azo/MgCl_2_/AC FSC near at 116% at different bending angles (Figure 5d), indicating that the P‐Azo/MgCl_2_/AC FSC has excellent mechanical stability. Under different bending angles, the internal resistance (*R*_s_) of the supercapacitor varies between 6.5 and 7.5 Ω, indicating that the interface resistance between the gel electrolyte and the positive and negative electrodes remains unchanged. This result shows that the strong adhesion between the polyacrylamide gel electrolyte and the positive and negative electrodes is the main reason for its stable mechanical properties. It is worth mentioning that the supercapacitor was subjected to 80 000 bending cycles using a bender (Figure S11, Supporting Information), and the supercapacitor still has capacitance retention of 90.7% (Figure 5e), indicating that it has excellent fatigue resistance.

### 2.4 Preparation of Photo‐Rechargeable Supercapacitors

**Figure 6:**
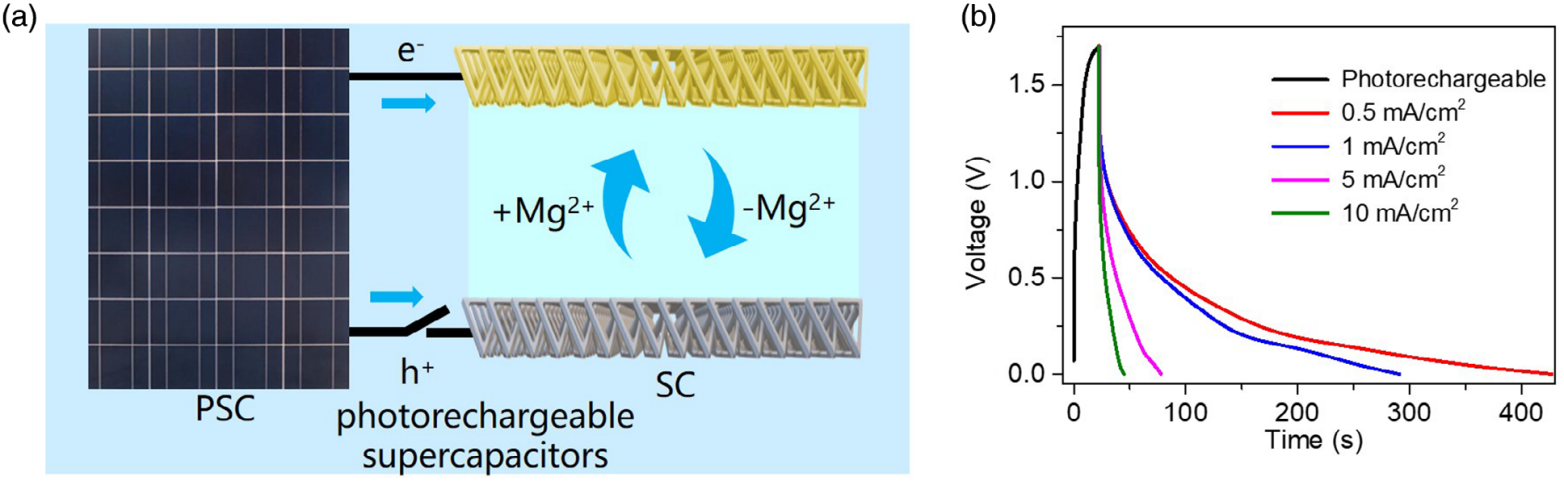
a) The working principle of photo‐rechargeable supercapacitors, and b) the GCD curves of photo‐rechargeable supercapacitors.

Based on the excellent electrochemical performance of the P‐Azo/MgCl_2_/AC FSC, the anode and cathode of the PSC are connected to the anode and cathode of the supercapacitor, respectively, to prepare photo‐rechargeable supercapacitors (**Figure** **6**a). Under illumination, the electrons and holes generated by the PSC flow into the cathode and anode of the supercapacitor to realize charge storage. The obtained PSC (FA_0.91_Cs_0.09_PbI_3_) exhibited an open‐circuit voltage of 1.6 V, a short‐circuit current of 11.53 mA cm^−2^, and a power conversion efficiency (PCE) of 14.7% (Figure S12, Supporting Information). To evaluate the photoelectric conversion performance of the photo‐rechargeable supercapacitor, the charging curve of the supercapacitor was measured under sunlight at an intensity of 100 mW cm^−2^, and then the light source was turned off to obtain the discharge curves under different current densities in the dark. When the discharge current of the P‐Azo/MgCl_2_/AC device is 10 mA cm^−2^, the charge time and discharge time are relatively close (Figure 6b), and the energy stored in the FSC is 52.89 mW h cm^−2^. When the discharge current of the FSC is 0.5 mA cm^−2^, the discharge time is much higher than the charging time, and the energy stored in the FSC is 47.77 mW h cm^−2^. This shows that the FSC enables fast charge storage at high current densities.

The formula for calculating the overall conversion efficiency of a photo‐rechargeable supercapacitor is as follows^[^ ^1^ ^]^

(4) $$\eta_{\text{overall}}=\frac{E_{\text{output}}}{E_{\text{light}}}=\frac{E_{\text{sc}}\times A_{\text{sc}}}{P_{i}\times \Delta t\times A}\times 100\%$$

where *E*_light_ is the light input energy, *E*_output_ is the energy stored in the supercapacitor, *E*_sc_ is the areal energy density of the supercapacitor, *A*_sc_ is the effective area of the supercapacitor, *P*_*i*_ is the light intensity (100 mW cm^−2^), Δ*t* is the light charging time, and *A* (cm^2^) is the effective light area of the solar cell. At a discharge current density of 1 mA cm^−2^, the overall conversion efficiency of the photo‐rechargeable supercapacitor is 7%, which is comparable to that of similar photo‐rechargeable supercapacitor reported so far.^[^ ^46^ ^]^ In the current density range of 0.5–10 mA cm^−2^, the overall conversion efficiency of the photo‐rechargeable supercapacitors varies between 5.2% and 7% (Figure S13, Supporting Information), indicating that supercapacitors have good charge‐storage capacity. This excellent electrochemical performance of photoelectric storage is due to the high energy density of these supercapacitors. These photo‐rechargeable supercapacitors show great application potential in the fields of flexible wearable electronic devices, satellites, hybrid electric vehicles, robots, notebook computers, drones, and distributed photovoltaic power generation.

## 3 Conclusion

We first apply P‐diaminoazobenzene (P‐Azo) as a new type of organic electrode material to prepare high‐energy‐density, high‐power‐density, and fatigue‐resistant all‐around high‐performance asymmetric flexible supercapacitors. The molecular structure of P‐Azo not only increases the conductivity of organic molecules but also makes it have a high energy density. P‐Azo is assembled with activated carbon into asymmetric flexible supercapacitors to achieve high areal specific capacity (1195.875 mF cm^−2^ at 0.1 mA cm^−2^) and energy density (425.2 mW h cm^−2^ at 80 mW cm^−2^, 10.44 mW h cm^−2^ at 8000 mW cm^−2^). In addition, the flexible supercapacitor still has stable capacitance retention after bending at different bending angles and 80 000 cycles. Supercapacitors and perovskite submodules are coupled to prepare a photo‐rechargeable supercapacitor to achieve a 7% overall energy conversion efficiency. Therefore, this flexible supercapacitor paves a practical route for powering future wearable electronics.

## 4 Experimental Section

4.1

4.1.1

##### Preparation of Organic Electrodes

P‐diaminoazobenzene, acetylene black, and polyvinylidene fluoride were uniformly mixed in *N*‐methyl pyrrolidone in a ratio of 7:2:1 by mass, then coated onto carbon cloth, and dried in a drying oven at 70 °C. The loading of active material in the electrode was 2 mg cm^−2^.

##### Fabrication of Flexible Supercapacitors

First, 4 g of MgCl_2_ was dissolved in 10 g of deionized water. Then, 4 g of acrylamide and 100 μL of photoinitiator 2‐hydroxy‐2‐methyl‐1‐phenyl‐1‐acetone were added to it. The polyacrylamide ion gel electrolyte was obtained by free‐radical polymerization under ultraviolet irradiation for 5 min.

The organic electrode was used as the negative electrode, the activated carbon was used as the positive electrode, and the polyacrylamide ion gel was used as the electrolyte, all of which were assembled into an all‐solid‐state flexible supercapacitor.

##### Preparation of Photo‐Rechargeable Supercapacitor

Fabrication of PSCs was performed by our reported method.^[^ ^47^ ^]^ To form the photo‐rechargeable supercapacitor, the cathode of the PSC was connected to the cathode of the supercapacitor with a wire, and the anode of the PSC was connected to the anode of the supercapacitor. An electrochemical workstation was used to test the charge–discharge curve of the supercapacitor.

##### Electrochemical Performance Test

The electrochemical performance of the electrodes was tested using a three‐electrode system with Pt as the counter electrode, Ag/AgCl as the reference electrode, and either 1 mol L^−1^ HCl, 1 mol L^−1^ LiCl, 1 mol L^−1^ NaCl, 1 mol L^−1^ MgCl_2_, 1 mol L^−1^ ZnCl_2_, or 1 mol L^−1^ of AlCl_3_ as the electrolyte. CV curves, GCD curves, and EIS curves of the test electrode were obtained. The electrochemical performance of the all‐solid‐state flexible supercapacitor was tested using a two‐electrode system.

The areal specific capacity of the electrodes was calculated using Equation (5)

(5) $$C=\frac{I\Delta t}{A\times \Delta V}$$

where *C* is areal specific capacity, *I* is the charge–discharge current of the electrode, Δ*t* is the charge–discharge time, Δ*V* is the electrochemical window of the electrode, and *A* is the area of the electrode.

The areal energy densities (*E*) of the electrodes were calculated using Equation (6)

(6) $$E=\frac{C\times \Delta V^{2}}{7.2}$$

The areal power densities (*P*) of the electrodes were calculated using Equation (7)

(7) $$P=\frac{E}{\Delta t}\times 3600$$

## Conflict of Interest

The authors declare no conflict of interest.

## Supporting information

Supplementary Material

## Data Availability

The data that support the findings of this study are available from the corresponding author upon reasonable request.
