# Supplementary material for: An Azo‐Based Electrode for All‐Around High‐Performance Flexible Supercapacitors
Source: Small Sci. 2023 Mar 9;3(5):2200101. doi: 10.1002/smsc.202200101 (PMC11935807; doi:10.1002/smsc.202200101)
Supplement: Supplementary file 1 — Supplementary Material [file SMSC-3-2200101-s001.pdf]

Azo-based electrode for all-around high-performance flexible supercapacitors

Haoxiang Zhang,[1,2] Minyong Du,[1,2] Xinxin Xing,[1] Hui Wang,[1,2]* Kai Wang, [1,2]* and Shengzhong (Frank) Liu[1,2,3]*

[1] Dalian National Laboratory for Clean Energy, Dalian Institute of Chemical Physics, Chinese Academy of Sciences, Dalian 116023, Liaoning, China;

[2] Center of Materials Science and Optoelectronics Engineering, University of Chinese Academy of Sciences, Beijing 100049, P. R. China.

[3] Key Laboratory of Applied Surface and Colloid Chemistry, Ministry of Education, Shaanxi Key Laboratory for Advanced Energy Devices, Shaanxi Engineering Lab for Advanced energy Technology, Institute for Advanced Energy Materials, School of Materials Science and Engineering, Shaanxi Normal University, Xi'an 710119, China

E-mail:hwang1606@dicp.ac.cn

E-mail: wangkai@dicp.ac.cn

E-mail: szliu@dicp.ac.cn

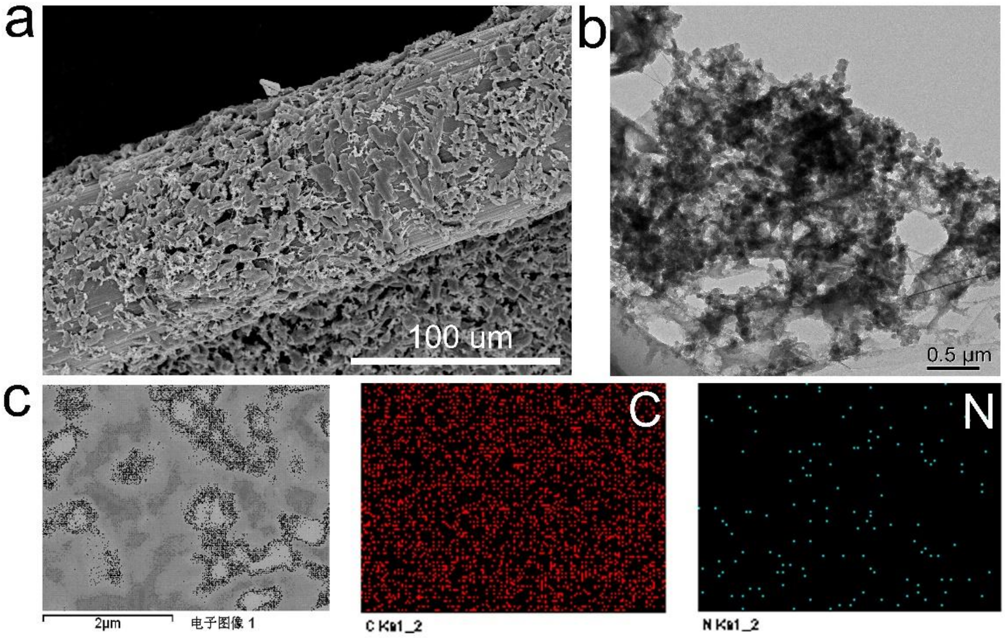

Figure S1. (a) SEM images of P-Azo electrode, (b) TEM images of P-Azo electrode, (c) SEM-EDX elemental mapping images of P-Azo electrode.

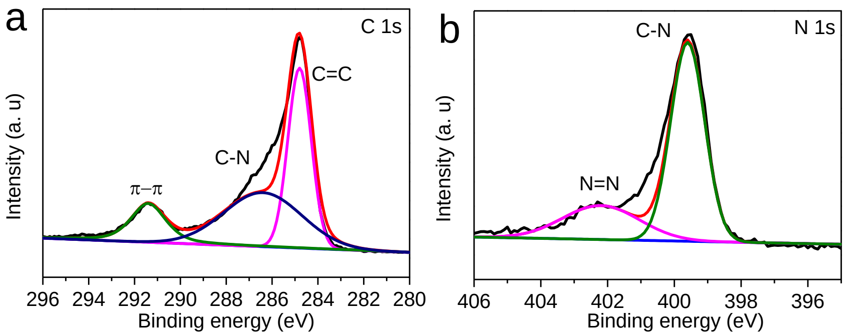

Figure S2. The P-Azo electrode high-resolution XPS spectra of (a) C 1 s and (b) N 1 s.

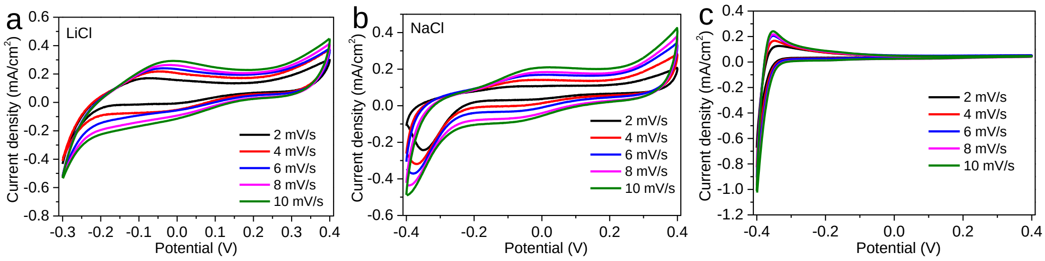

Figure S3. CV curves of P-Azo in (a) LiCl, (b) NaCl and (c) $AlCl_3$ solution

Table S1. Fitting equation of b value of P-Azo in different solutions

	Curve fitting	R^2
HCl	log*i*=0.67log*v*-3.83	0.997
$MgCl_2$	log*i* =0.57 log*v* - 3.79	0.927
$ZnCl_2$	log*i* =0.83 log*v* -3.9	0.999

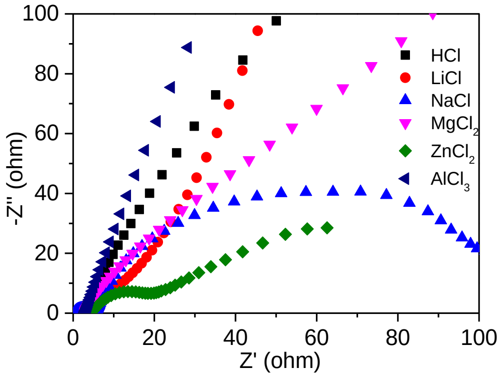

Figure S4. EIS curves of P-Azon in different solutions

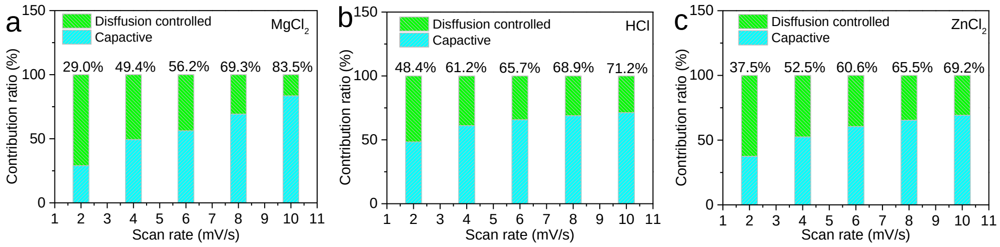

Figure S5. Capacitance contribution of P-Azo in (a) $MgCl_2$, (b) HCl and (c) $ZnCl_2$ solutions

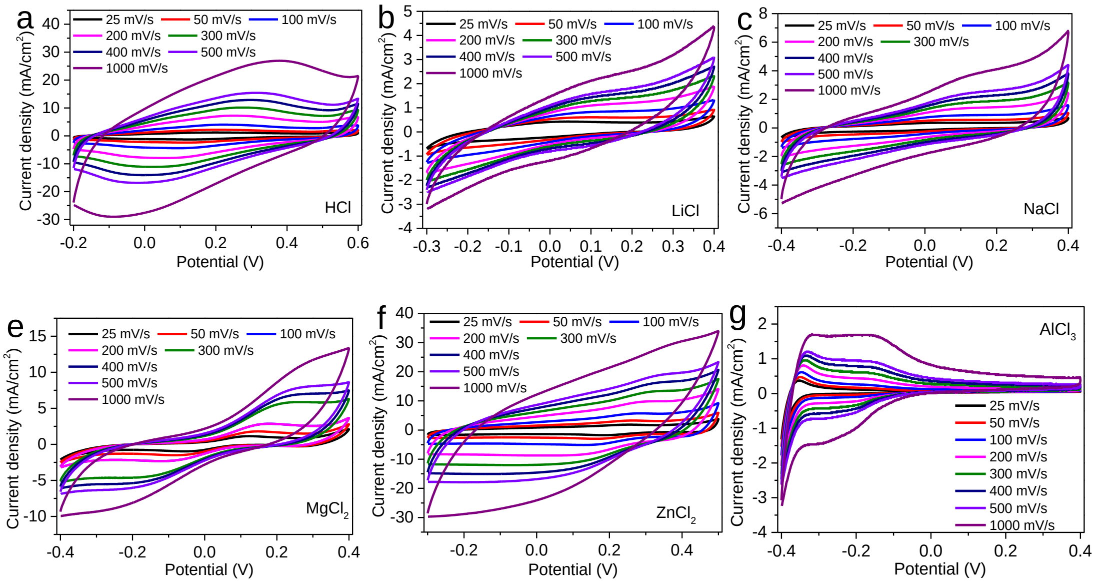

Figure S6. CV curves of P-Azo at (a) 1mol/L HCl, (b) 1mol/L LiCl, (c) 1mol/L NaCl, (d) 1mol/L $MgCl_2$, (e) 1mol/L $ZnCl_2$, (f) of 1mol/L $AlCl_3$.

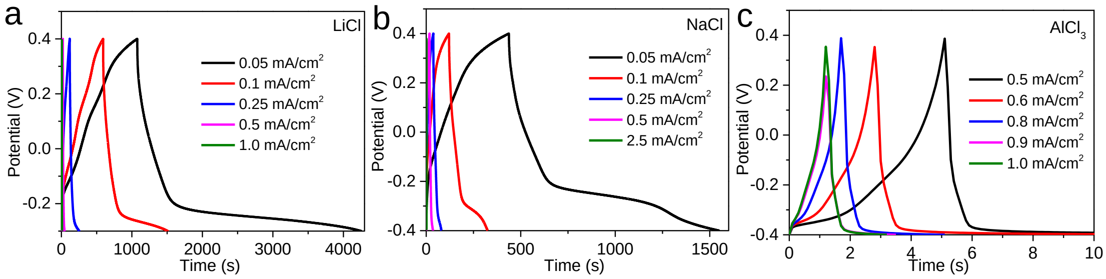

Figure S7. GCD curves of P-Azo in (a) LiCl, (b) NaCl and (c) $AlCl_3$ solutions

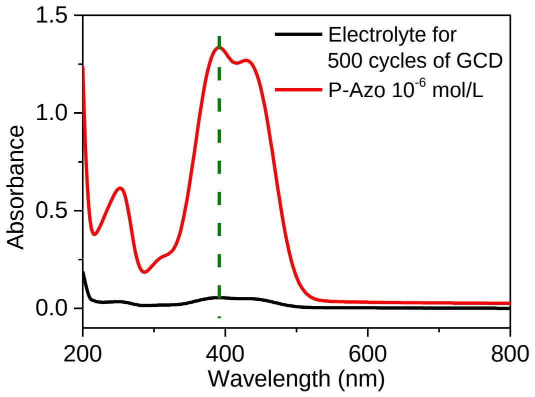

Figure S8. The UV absorption spectrum of the P-Azo after 500 cycles of charge and discharge in the electrolyte and the UV absorption spectrum of 10 mol/L P-Azo.

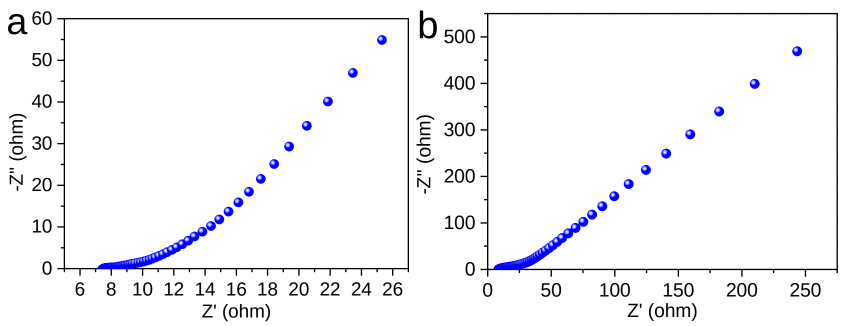

Figure S9. EIS curve of (a) P-Azo/$MgCl_2$/AC FSC and (b) P-Azo/$ZnCl_2$/AC FSC

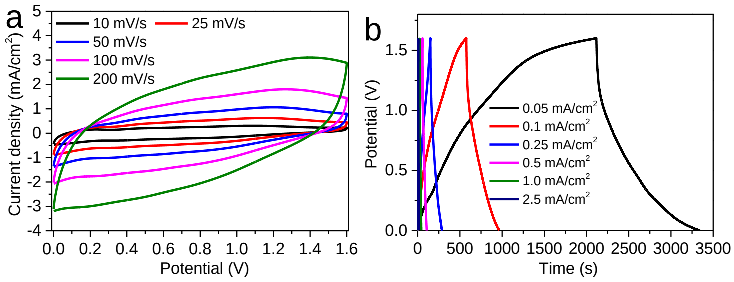

Figure S10. (a) CV curves and (b) GCD curves of P-Azo/$ZnCl_2$/AC FSC.

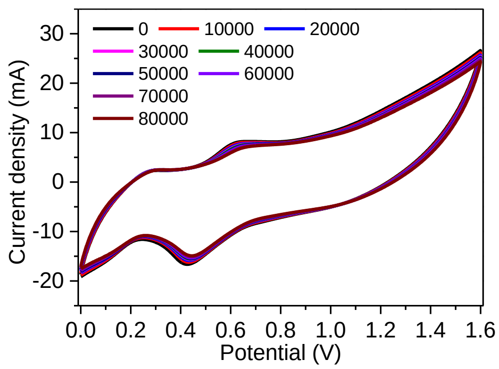

Figure S11. The CV curves of P-Azo/$ZnCl_2$/AC FSC bent 80000 times

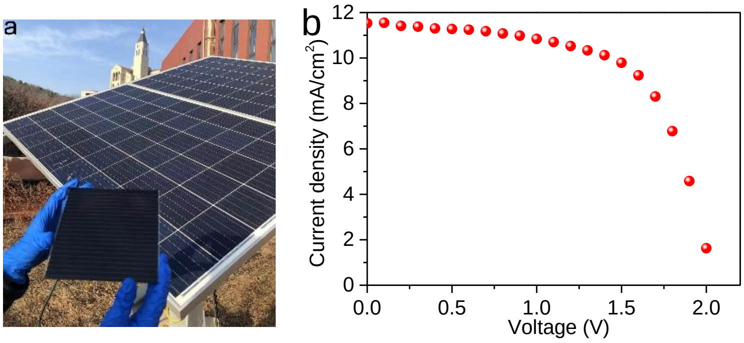

Figure S12. (a) photo of PSC, (b) J-V curve of PSC.

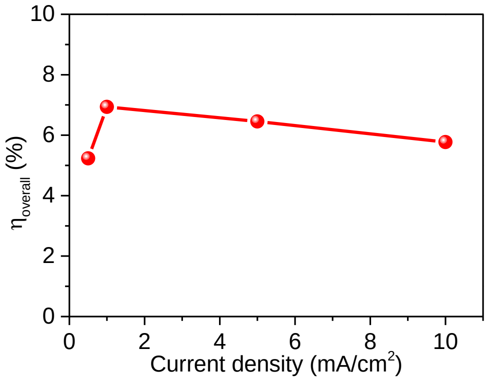

Figure S13. The overall conversion efficiency of photorechargeable supercapacitors as a function of current density.
